# Identification of *Staphylococcus* species isolated from preputium of Aceh cattle based on 16S rRNA gene sequences analysis

**DOI:** 10.14202/vetworld.2019.1540-1545

**Published:** 2019-10-08

**Authors:** Muhammad Hambal, Masda Admi, Safika Safika, Wahyu Eka Sari, Teuku Reza Ferasyi, Dasrul Dasrul, Ummu Balqis, Darmawi Darmawi

**Affiliations:** 1 Laboratory of Parasitology, Faculty of Veterinary Medicine, Universitas Syiah Kuala, Banda Aceh, Aceh, Indonesia; 2 Laboratory of Microbiology, Faculty of Veterinary Medicine, Universitas Syiah Kuala, Banda Aceh, Aceh, Indonesia; 3 Department of Mathematics and Applied Sciences, Universitas Syiah Kuala, Darussalam, Banda Aceh, 23111, Indonesia; 4 Department of Veterinary Infectious Diseases and Veterinary Public Health, Faculty of Veterinary Medicine, IPB University, Jl. Agatis Dramaga, Bogor, 16680, West Java, Indonesia; 5 Laboratory of Research, Faculty of Veterinary Medicine, Universitas Syiah Kuala, Banda Aceh, Aceh, Indonesia; 6 Laboratory of Veterinary Public Health, Faculty of Veterinary Medicine, Universitas Syiah Kuala, Banda Aceh, Aceh, Indonesia; 7 Laboratory of Reproduction, Faculty of Veterinary Medicine, Universitas Syiah Kuala, Banda Aceh, Aceh, Indonesia; 8 Laboratory of Pathology, Faculty of Veterinary Medicine, Universitas Syiah Kuala, Banda Aceh, Aceh, Indonesia; 9 Laboratory of Biomedical Science, Faculty of Public Health, University of Teuku Umar, Meulaboh, Aceh, Indonesia

**Keywords:** 16S rRNA gene, Aceh cattle, phylogenetic tree, polymerase chain reaction, *Staphylococcus pasteuri*

## Abstract

**Aim:**

This research aimed to identify *Staphylococcus* species isolated from preputial swabs of healthy Aceh cattle, based on 16S ribosomal RNA gene analysis.

**Materials and Methods:**

The bacterium was isolated from preputial swabs of healthy Aceh cattle. The total DNA from the isolated bacteria was extracted using the Genomic DNA Mini Kit followed by polymerase chain reaction (PCR) amplification of the 16S rRNA gene. The product of PCR amplification was then sequenced and aligned to the known sequences in the GenBank database by multiple alignments and was also analyzed by bioinformatics software to construct a phylogenetic tree.

**Results:**

The results revealed that the bacterial isolate 3A had genetically closed relation to *Staphylococcus pasteuri* with <97% maximum identity. Data derived from the phylogenetic tree revealed that the bacterial isolate 3A was also related to *Staphylococcus warneri*, yet, it shows a different evolutionary distance with the ancestors *(S. pasteuri)*.

**Conclusion:**

The results of this research suggested that the bacterium 3A, isolated from preputial swabs of healthy Aceh cattle, is a *Staphylococcus* species.

## Introduction

There is a permanent need for dairy farms and their development in Indonesia, as it is inseverable and integral part of the farming system. There are about 14.90 million cattle, contributing to 4.80% of the national Growth Domestic Product [1]. Aceh cattle are one of several indigenous Indonesian cattle and are acknowledged as an Indonesian domestic breed with many positive characteristics for tropical climates. The population of Aceh cattle was recorded to be more than 511 thousand in 2015 and is concentrated in the Aceh Province [2]. Aceh cattle play a major role in beef production, so they are usually referred to as beef cattle, besides being able to produce milk. They are also the principle animals for draft work in rice fields. As such, Aceh cattle must be conserved by focusing on diseases caused by animal pathogens to ensure the availability of adequate supplies of Aceh cattle and their products in the future.

Therefore, in this research, we focused on pathogenic bacteria which are a potential risk to reproduction organs, causing widespread disease, especially during Aceh cattle breeding. Microbial contamination affects motility, morphology, and various semen quality parameters [3]. Preputium infections caused by bacteria have been a primary concern for cattle breeders, as they can affect the reproductive performance of cattle. During the breeding season, the prepuce bacteria may be transmitted to other cattle, playing an important role in serious diseases. Various types of bacteria present in the soil, bedding, and manure can cause contamination with entry through the preputial orifice into the preputium cavity of cattle; this can occur frequently. In previous studies, several authors have reported potential sources of preputium infection [4-6].

There are numerous studies regarding *Staphylococcus pasteuri* as a potential pathogen found in various environmental conditions. The spread of *S. pasteuri* in plant food environments and animals has been reported by several authors and their studies described that vegetables [7,8], and leafy vegetables, including perilla leaf, chicory, and lettuce from local markets in South Korea [8], pig farms in the Netherlands [9], and bovine mastitis from Brazilian dairy herds [10] were contaminated or caused by *S. pasteuri*. In our previous investigation of cellulolytic *Enterobacte* r [11], and cellulolytic *Bacillu* s [12], both bacteria were found in the rumen of Aceh cattle. Recently, in the preputial swabs of Aceh cattle, we found prevalence of 8.0% of *Escherichia fergusonii* identified by phylogenetic 16S rRNA analysis [13]. The 16S rRNA gene has highly conserved sequences within species and between species of the same genus so that this gene can be used as the common tool for the speciation or identification of bacteria.

Thus, 16S rRNA gene analysis of bacteria from preputial swabs of clinically healthy Aceh cattle needs to be conducted. Our research goal was to identify the bacterium isolate 3A isolated from preputial swabs of healthy Aceh cattle, based on molecular identification of the bacterial 16S rRNA gene by bioinformatic analysis and construction of a phylogenetic tree.

## Materials and Methods

### Ethical approval

All procedures of this research involving animal care were carried out in accordance with local government guidelines. They were approved by the Animal Ethics Committee of Faculty of Veterinary Medicine, Universitas Syiah Kuala, Aceh, Indonesia (approval No. 014/KEPH-C/III/2017).

### Preparation of animals

Aceh cattle are indigenous animals in Aceh, which are unique in that they are smaller in size than cattle from other regions. The cattle used in this research were male cattle more than 2 years old and in good clinical health. Preputium samples were obtained from 50 healthy Aceh cattle, kept in Indrapuri Breeding Aceh and Forage Center of Aceh Cattle, Aceh Besar, Indonesia, under sterile, hygienic conditions. The external preputium of the Aceh cattle was sterilized and rinsed with 0.9% sodium chloride.

### Bacterial isolate

The bacterial isolate 3A was obtained from the collection of Microbiology Laboratory, Faculty of Veterinary Medicine, Universitas Syiah Kuala. This isolate was isolated from preputial swabs samples and cultured on MacConkey agar (Difco Laboratories, USA), then incubated at 37°C for 24 h. A total of 1.5 mL of bacterial culture isolate 3A were used for DNA isolation.

### DNA isolation

Total genomic DNA from bacterium isolated from preputial swabs of healthy Aceh cattle was isolated using a gDNA Presto^™^ Bacteria Mini kit (Geneaid) with a slightly modified protocol [14]. Purified total genomic DNA (50 µL, ~200 µg/mL) was eluted and used as a template for polymerase chain reaction (PCR) amplification following the protocol [11].

### PCR amplification of 16S rRNA gene

The DNA was amplified through PCR using C1000 Thermal Cycler (BIO-RAD) as follows: A DNA template of 30 ng bacterial isolate 3A was added to a total reaction mixture (25 µL total) containing 12.5 µL of *Taq* polymerase master mix (KAPA Biosystems, MA, USA), primer forward (5’-AGAGTTTGATC(A/C)TGGCTCAG-3’) and primer reverse (5’-GGTTAC(G/C)TTGTTACCTGCCGGA-3’) [15], and 10 pmol of each primer. The amplification was performed with initial denaturation step at 95°C for 5 min, followed by 30 cycles of amplification [16] 95°C for 1 min denaturation, 50°C annealing for 30 s, and 72°C extension for 2 min with a final extension step at 72°C for 10 min. The ~1500 bp PCR products were visualized on a 1.2% (w/v) agarose gel by electrophoresis in 1× Tris-Acetate-EDTA buffer pH 8.3 (40 mM Tris-HCl, 40 mM acetate, and 1.0 mM EDTA) and observed with the Gel Doc XR+ System (BIO-RAD).

### 16S rRNA gene sequencing, analysis of bioinformatics, and construction of the phylogenetic tree

The amplification product was directly sequenced using a DNA sequencer (Macrogen Inc., Korea). The 16S rRNA gene sequences data from isolate 3A were compared to the GenBank database using the Basic Local Alignment Search Tool (BLAST) software (blastn) available from the National Center for Biotechnology Information (NCBI) (http://www.ncbi.nlm.nih.gov/). The 16S rRNA sequence was analyzed and aligned using the ClustalW program, and the phylogenetic tree was constructed using MEGA 5.05 software (https://www.megasoftware.net/,) [12,13,17], based on the neighbor-joining tree method [18] and refers to the model p-distance, with bootstrap 1000× [19]. An outgroup for phylogenetic analysis used in this study is an *Escherichia col* i (Enterohemorrhagic *E. coli*).

## Results

**Figure-1:**
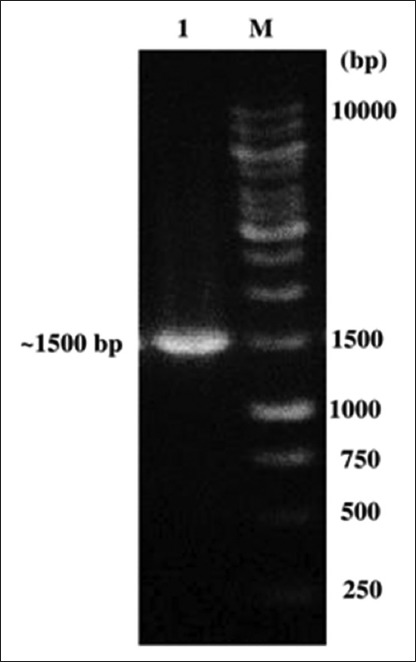
Polymerase chain reaction amplification product on Agarose gel (1%) (~1500 bp). Lane 1: Bacterial isolate 3A; lane 2: Marker 1 Kb.

The bacterial isolate 3A isolated from preputial swabs of Aceh cattle produced a single band with expected molecular size of ~1500 bp (Figure-1). Based on blast results, bacterial isolate 3A was closely related to *Staphylococcus* species with 95–96% maximum identity. The similarity of bacterial isolate 3A with *S. pasteuri* strain ATCC 51129 (Accession No. NR_114435.1) was 96% maximum identity, with an E-value 0.0 (Table-1).

**Table 1:** Percent similarity the sequences of 16S rRNA bacterial isolate 3A to *Staphylococcus* species.

References (GenBank)	% similarity	E-value	Accession no.
*Staphylococcus pasteuri* strain ATCC 51129	96	0.0	NR_114435.1
*Staphylococcus pasteuri* strain ATCC 51129	96	0.0	NR_024669.1
*Staphylococcus warneri* strain AW 25	96	0.0	NR_025922.1
*Staphylococcus devriesei* strain KS-SP	95	0.0	NR_116627.1
*Staphylococcus epidermidis* strain NBRC 100911	95	0.0	NR_113957.1
*Staphylococcus caprae* strain DSM 20608	95	0.0	NR_119252.1
*Staphylococcus capitis* spp. urealyticus strain MAW 8436	95	0.0	NR_027519.1
*Staphylococcus epidermidis* strain Fussel	95	0.0	NR_036904.1
*Staphylococcus lugdunensis* strain ATCC 43809	95	0.0	NR_024668.1
*Staphylococcus caprae* strain ATCC 35538	95	0.0	NR_0244665.1

**Figure-2:**
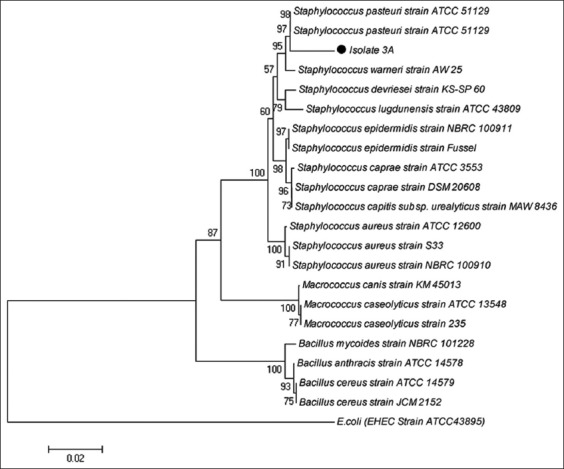
Figure-2: A phylogenetic tree indicating the position of Aceh cattle preputial swab isolate 3A.

The bacterial isolate 3A had the highest similarities with *S. pasteuri* at 96% maximum identity, followed by *Staphylococcus warneri* with 96% maximum ­identity, then *Staphylococcus devriesei*, *Staphylococcus epidermidis*, *Staphylococcus caprae*, *Staphylococcus capitis*, and *Staphylococcus lugdunensis*, with 95% maximum identity (Table-1). The phylogenetic tree showed that bacterial isolate 3A was closely related to *S. pasteuri* strain ATCC 51129 and separated from the outgroup cluster negative bacteria (*E. coli*) (Figure-2).

## Discussion

Cases of reproductive organ disorders in cattle are increasing over recent years. These disorders, such as abortions and infertility in cattle, are important problems in the cattle industry; they can be caused by several various factors, such as physical, biological, chemical, and environmental agents [20]. The infectious agents in animal reproductive organs, such as bacteria, are known to have a direct effect on cattle reproductive health. Its complexity can be increased by preputial bacteria which can ultimately determine the overall animal’s health. In previous research, each animal appears to have different bacterial communities. For example, Yoo [21] reported that pathogenic bacteria such as *Brucella abortus*, *Leptospira interrogans*, *Campylobacter fetus*, *Salmonella* spp., and *Listeria monocytogenes* are involved in reproductive disorders in bovines, including abortion and sterility in cattle. In addition, bovine brucellosis due to *B. abortus* results in infertility in male cattle and abortion in female cattle which are infected and release bacteria in their semen [22,23]. Haapala *et al*. [24] reported semen as a source of *Mycoplasma bovis* mastitis in dairy farms. *Staphylococcus* spp. have also been reported from the preputium of healthy dogs [25]. Different microorganisms that contaminate semen samples of cattle bulls include *Staphylococcus* spp., *Micrococcus* spp., *E. coli*, *Pseudomonas* spp., *Corynebacterium* spp., *Proteus* spp., *Klebsiella* spp., *Bacillus* spp., and *Streptococcus* spp. [6].

DNA sequencing techniques are becoming less expensive and increasingly popular in a clinical environment [26]. An analysis of 16S rRNA is frequently used in molecular identification and construction of phylogenetic relationship among prokaryotes because the 16S rRNA gene is well conserved in bacteria and is large enough for bioinformatics purposes [27]. In 1994, two strains were considered to belong to novel species if they share the 16S rRNA gene sequence similarity lower than 97% and to discriminate two genera if this value was lower than 95% [28]. The phylogenetic relationship of taxa in phylogenetic trees constructed from the largely conserved 16S rRNA gene sequences confirmed the largely morphological characteristics and classic phenotypes used for classification schemes for species of the genus *Staphylococcus* [26,29-31]. Based on 16S rRNA gene analysis, it is reported here that bacterial isolate 3A is indicated as a novel species with <97% maximum identity, E-value 0.0, and it belonged to *Staphylococcus* spp. Based on the phylogenetic tree, the bacterial isolate 3A was most closely related to *S. pasteuri* strain ATCC 51129.

*S. pasteuri* strain ATCC 51129 was first reported by Takahashi *et al*. [30]. Based on phylogenetic analysis, this species was closely related to *S. epidermidis*, *S. lugdunensis*, *Staphylococcus haemolyticus*, *S. capitis*, *Staphylococcus saccharolyticus*, *S. caprae*, *Staphylococcus hominis*, and *S. warneri*. This report supports the results of the phylogenetic tree analysis shown in this study (Figure-2). The characteristics of *S. pasteuri* are that it is cocci, nonmotile, nonsporulating, Gram-positive, with a diameter of 0.5-1.5 µm, occurring singly, and the colonies are glistening, smooth, and raised with slightly elevated centers and regular edges. *S. pasteuri* spp. nov. has been successfully isolated from animals, food specimens, and humans [29].

The findings of the present study are in agreement with the report of various authors which indicated that healthy animals can become subclinical carriers, which can act as reservoirs to transmit *S. pasteuri*. The study by Tulinski *et al*. [9], conducted on pig farms in the Netherlands, showed that *S. pasteuri* bacteria appeared in nasal swabs of pigs. Regecová *et al*. [32] reported that 3.8% of sea fish were positive for *S. pasteuri*. Another investigation in Brazilian dairy herds using milk samples from bovine mastitis cases reported the incidence of *S. pasteuri* as 3.3% [10]. According to epidemiological analysis, Savini *et al*. [33] documented that *S. pasteuri* can cross from mammals and lampreys to man.

*S. pasteuri* is not only crucial for veterinary public health but also is as an agent of human diseases. It has been identified by 16S rRNA analysis as causing a bacteremia episode in a leukemia patient [33]. *S. pasteuri* bacteria are emerging as an agent of nosocomial infections. They are known to contaminate wastewater and drinking water [34], blood products [35], and platelet units from an adult 1^st^-time donor [33]. Furthermore, *S. pasteuri* was the nosocomial bacteria that were isolated from platelet concentrates [36], in a patient with leukemia [33], from an apheresis platelet product [33], from the community and hospital environments in Thailand [37], hotel rooms [38], and non-health-care environments in London. *S. pasteuri* has also frequently been found in the food and associated environments, namely, food contact surfaces and hands of food care workers [39].

In particular, *S. pasteuri* may act as a reservoir to transfer genes from coagulase-negative staphylococci to other staphylococcal species. Several studies have demonstrated that antibiotic-resistant genes could be transferred between staphylococcal species. *S. pasteuri* have been reported to be resistant to several antibiotics, such as methicillin [9,37], streptomycin, and other various antibiotics [38]. *S. pasteuri* shows resistance to various antimicrobial agents, so it causes difficulties in treatment strategies.

Importantly, although *S. pasteuri* considered a bacterial pathogen under certain circumstances in animals and humans, the bacterium may produce bacteriocin, a ribosomally synthesized peptide that has great potential as an alternative antimicrobial agent. Hong *et al*. [7] suggested that products released by *S. pasteuri* RSP-1 isolate from leafy vegetables may be utilized to effectively inhibit the growth of *Staphylococcus aureus*. Recently, Hong *et al*. [7] successfully characterized and purified pasteuricin produced by *S. pasteuri* RSP-1 that act as ­antimicrobial activity against Gram-positive bacteria including methicillin-resistant *S. aureus*.

*S. pasteuri* can cross from one species to another species, which may play an important role as in it being both a veterinary and human pathogen. Asymptomatic cattle carriers serve as a reservoir for transmission of *S. pasteuri* among ruminant livestock in the cattle farm environment. Host-adapted *S. pasteuri* can result in endemic disease that is maintained on a farm by carrier animals shedding in the urine and/or semen, indicating significant sources of cross-contamination between cattle. *S. pasteuri* has already been found in preputium, potentially allowing the bacterium to be spread and can thus pose a significant risk to veterinary public health in cattle breeding farm. To avoid such contaminations, the proper and accurate analysis of the reproductive tract, notably the preputium, is important. The ability of *S. pasteuri* to survive in the preputium shows the need for special care to be taken during the breeding season and collection of semen from cattle to prevent contamination. Moreover, the preputium of cattle should be recognized as a reservoir of some pathogens and as being of great importance to veterinary public health. The hygienic status of the preputium is important for preventing potential pathogenic bacteria transmission through natural mating conditions or artificial insemination.

## Conclusion

The bacterial isolate 3A from preputial swabs of healthy Aceh cattle is indicated to be a *Staphylococcus* species, based on analysis of 16S rRNA gene sequences. The phylogenetic tree showed that this bacterial isolate was related to *S. pasteuri*, and clustered together with *S. warneri*, *Staphylococcus devriesei*, *S. lugdunensis*, *S. epidermidis*, *S. caprae*, *S. aureus*, and *S. caseolyticus*.

## Authors’ Contributions

DD and MH conceptualized and designed this research. The research was carried out by MA, SS, DDasrul, and TRF. SS and WES analyzed the data and result. MH, WES, UB, and DD drafted, revised and finalized the manuscript. All authors read and approved the final manuscript.
